# Decreased neurofilament light chain levels in estriol‐treated multiple sclerosis

**DOI:** 10.1002/acn3.51622

**Published:** 2022-06-29

**Authors:** Rhonda Voskuhl, Jens Kuhle, Prabha Siddarth, Noriko Itoh, Kevin Patel, Allan MacKenzie‐Graham

**Affiliations:** ^1^ UCLA Multiple Sclerosis Program, Department of Neurology David Geffen School of Medicine at the University of California Los Angeles California USA; ^2^ Neurologic Clinic and Policlinic, Departments of Medicine, Biomedicine and Clinical Research University Hospital Basel, University of Basel Basel Switzerland; ^3^ Jane and Terry Semel Institute for Neuroscience and Human Behavior University of California Los Angeles California USA; ^4^ Ahmanson‐Lovelace Brain Mapping Center, Department of Neurology David Geffen School of Medicine at the University of California Los Angeles California USA

## Abstract

Estrogens have neuroprotective actions depending on estrogen type, dose, and timing in both preclinical models and in women during health and disease. Serum neurofilament light chain is a putative biomarker of neurodegeneration in multiple sclerosis, aging, and other neurodegenerative diseases. Here, oral treatment with an estrogen unique to pregnancy (estriol) using an 8 mg dose to induce a mid‐pregnancy blood estriol level reduced serum neurofilament light chain in nonpregnant MS women at mean age of 37 years. This is consistent with estriol‐mediated protection from neuro‐axonal injury and supports the use of serum neurofilament light chain as a biomarker in MS.

## Introduction

Estrogens have neuroprotective properties in noninflammatory neurodegenerative conditions, which are dependent on estrogen type, dose, and timing.^1^, ^2^, ^3^, ^4^, ^5^ Estriol is made by the fetoplacental unit during pregnancy, with blood estriol levels increasing across the three trimesters.^6^ Beneficial effects of pregnancy and multiparity have been observed in women with multiple sclerosis (MS).^7^ Here, we determined serum neurofilament light chain (sNfL) levels in MS women treated with estriol at a dose aiming to induce serum levels consistent with pregnancy.^8^, ^9^ Sera were available from female subjects mean age 37 years (range 20–54) in a double‐blinded, placebo‐controlled, phase 2 trial of oral Estriol 8 mg versus Placebo treatment, each in combination with standard‐of‐care glatiramer acetate (GA).^8^ Whether there was a difference in sNfL levels in Estriol plus GA treated versus Placebo plus GA treated was determined, and associations of sNfL with MS disease measures were examined.

## Methods

### Design

Given the reduction in cerebral cortex atrophy in the Estriol + GA‐treated group compared to Placebo + GA at month 12,^8^, ^10^ this study extended biomarker analyses to NfL in serum. Subjects for this NfL biomarker study are the same as in the MRI biomarker study (Estriol + GA, *n* = 62 and Placebo + GA, *n* = 49),^10^ with all three timepoints (month 0, 6, and 12) included in NfL assessments. Flow diagrams tracking patients after initial enrollment are published.^8^, ^10^ There was a reduction in cerebral cortex atrophy at month 12 in Estriol + GA compared to Placebo + GA in the original paper that included all subjects.^8^ There was a very similar level of reduction in cerebral cortex atrophy at month 12 in Estriol + GA compared to Placebo + GA in the follow‐up paper that included a subset of subjects.^10^ Thus, the subset was not biased. Regarding later timepoints, MRIs were not done at month 18, and there was poor compliance at end of trial (month 24) documented by a significant decrease in estriol blood levels.^8^

### Assessment of estriol levels

Three hundred and twenty‐eight serum samples were collected longitudinally from 111 MS patients (62 treated with Estriol 8 mg + GA and 49 treated with Placebo + GA) at month 0, 6, and 12 of treatment and measured for both total and free estriol levels using ELISA kits (Total Estriol Elisa Kit #IB79124 and Free Estriol Elisa Kit #IB79123; American Research Products, Inc.; Belmont, MA). All samples were examined for estriol levels on the same experimental days, as described.^9^

### Assessment of NfL levels

Blood draws and MRIs were not done within 6 weeks of relapse. Serum NfL measurements were determined at the University Hospital Basel, as described,^11^, ^12^ using additional aliquots of the same 328 serum samples. All data were inspected for outliers and homogeneity of variance. Age‐adjusted serum NfL percentile scores were log‐transformed prior to analyses in order to satisfy distributional assumptions for parametric tests.

### Statistical analysis

Treatment groups (Estriol 8 mg + GA and Placebo + GA) were compared using *t*‐tests (continuous variables) or chi‐squared/Fisher's exact tests (categorical variables) on measures at baseline. Estriol levels and log‐transformed NfL percentile scores from all samples were analyzed using a mixed effects general linear model, as implemented in SAS PROC MIXED, including treatment group, time, and the interaction between time and treatment group. The significance of the interaction between time and treatment group was used to assess whether the groups differed in changes in estriol levels or NfL scores. Post hoc analyses determined the significance of within‐group changes. Whether changes in sNfL scores were associated with changes in clinical or imaging measures was determined using mixed effects models, including these measures as time‐varying predictors (one at a time). An interaction term with treatment group was included to determine whether the associations were significantly different between the Estriol plus GA group and the Placebo plus GA group.

The trial protocol was approved by each site's Human Subjects Protection Review Board, and informed consent was obtained from each subject.

## Results

The Estriol + GA group and the Placebo + GA group did not differ in baseline characteristics regarding age, ethnicity, relapses prior to enrollment, MS disease duration, EDSS scores, gadolinium‐enhancing lesion volumes, T2 lesion volumes, or cerebral cortex volumes,^8^, ^10^ Table 1.

**Table 1:** Baseline characteristics.

	All patients (*n* = 111)	Estriol + GA (*n* = 62)	Placebo + GA (*n* = 49)	*p* value
Age (years)				0.72
Mean ± SD	37.3 ± 7.5	37.4 ± 7.9	37.2 ± 7.1	
Median, IQR	37.7, 32.7–43.2	39.0, 32.7–43.0	36.2, 32.6–43.8	
Range	20.0–53.6	20.0–53.6	20.6–51.0	
Ethnicity				0.36^1^
Black	11 (9.9%)	8 (12.9%)	3 (6.1%)	
Caucasian	90 (81.1%)	48 (77.4%)	42 (85.7%)	
Hispanic	9 (8.1%)	6 (9.7%)	3 (6.1%)	
Other	1 (0.9%)	0 (0.0%)	1 (2.0%)	
MS duration (years)				0.92
Mean ± SD	3.1 ± 4.5	3.2 ± 4.3	2.9 ± 4.7	
Median, IQR	0.7, 0.3–4.3	0.85, 0.3–5.1	0.6, 0.4–3.6	
Range	0.1–24.3	0.1–16.4	0.1–24.3	
EDSS score				0.29
Mean ± SD	2.2 ± 1.1	2.3 ± 1.1	2.1 ± 1.1	
Median, IQR	2.0, 1.5–3	2.5, 1.5–3.0	2.0, 1.5–3.0	
Range	0–5.5	0–4.0	0–5.5	
Gd‐enhancing lesion volume (mL)				0.37
Mean ± SD	.065 ± .186	.088 ± .236	.035 ± .085	
Median, IQR	0, 0–.031	0, 0–.050	0, 0–.025	
Range	0–1.162	0–1.162	0–.403	
T2/FLAIR lesion volume (mL)				0.39
Mean ± SD	5.7 ± 7.2	6.0 ± 6.9	5.7 ± 7.7	
Median, IQR	3.1, 1.2–6.6	3.7, 1.4–7.0	2.7, 1.1–6.0	
Range	0.1–34.6	0.2–34.6	0.1–32.7	
Cerebral Cortex Volume (mL)				0.16
Mean ± SD	760.6 ± 40.7	755.6 ± 44.6	766.9 ± 34.6	
Median, IQR	761.7, 731.9–795.9	755.3, 724.1–783.9	765.2, 747.7–801.3	
Range	660.0–849.8	660.0–849.8	684.4–823.1	

Wilcoxon rank sum test for all others.

^1^ Chi‐square test.

Total and free estriol levels were higher in Estriol 8 mg + GA group compared to Placebo + GA group at both 6 and 12 months (Table 2). Serum estriol levels in Estriol 8 mg + GA group approximated those of late second trimester to early third trimester of pregnancy,^6^, ^13^ consistent with levels previously reported for oral Estriol 8 mg per day dosing.^8^, ^9^ Levels in Placebo + GA were at the lower limits of detection, as expected in nonpregnant women.

**Table 2:** Estriol levels.

	Estriol + GA (*n* = 62)	Placebo + GA (*n* = 49)	Between‐groups statistics^1^
Total estriol levels			
Month 0	1.97 (1.83)	1.82 (1.01)	*p* = 0.7
Month 6	11.21 (7.06)^2^	1.82 (1.44)	*p* < 0.0001
Month 12	10.29 (7.35)^2^	1.85 (1.11)	*p* < 0.0001
Free estriol levels			
Month 0	0.56 (0.39)	0.51 (0.38)	*p* = 0.5
Month 6	2.14 (1.95)^2^	0.45 (0.40)	*p* < 0.0001
Month 12	1.90 (1.47)^2^	0.48 (0.41)	*p* < 0.0001

Total and free estriol levels in serum expressed in ng/mL in Estriol + GA‐treated and Placebo + GA‐treated groups, each shown as means with standard deviations (SD). Total estriol levels at both 6 and 12 months were significantly different between the two treatment groups, with levels higher in Estriol 8 mg + GA treated compared to Placebo + GA treated. Free estriol levels in serum showed the same pattern of change over time and between groups, albeit with lower absolute values than total.

^1^ Mixed model analyses yielded a significant interaction between group and time for both total (*F*(2,109) = 51.80, *p* < 0.0001) and free (*F*(2,109) = 27.67, *p* < 0.0001) estriol levels. Statistics presented are post hoc analyses of between‐group differences.

^2^ The Estriol 8 mg + GA group demonstrated a significant increase in total estriol levels from baseline to 6 months (*t*(109) = 14.37, *p* < 0.0001) and from baseline to 12 months (*t*(109) = 12.88, *p* < 0.0001). The Placebo + GA group showed no change in estriol levels from baseline to 6 or 12 months.

**Figure 1:**
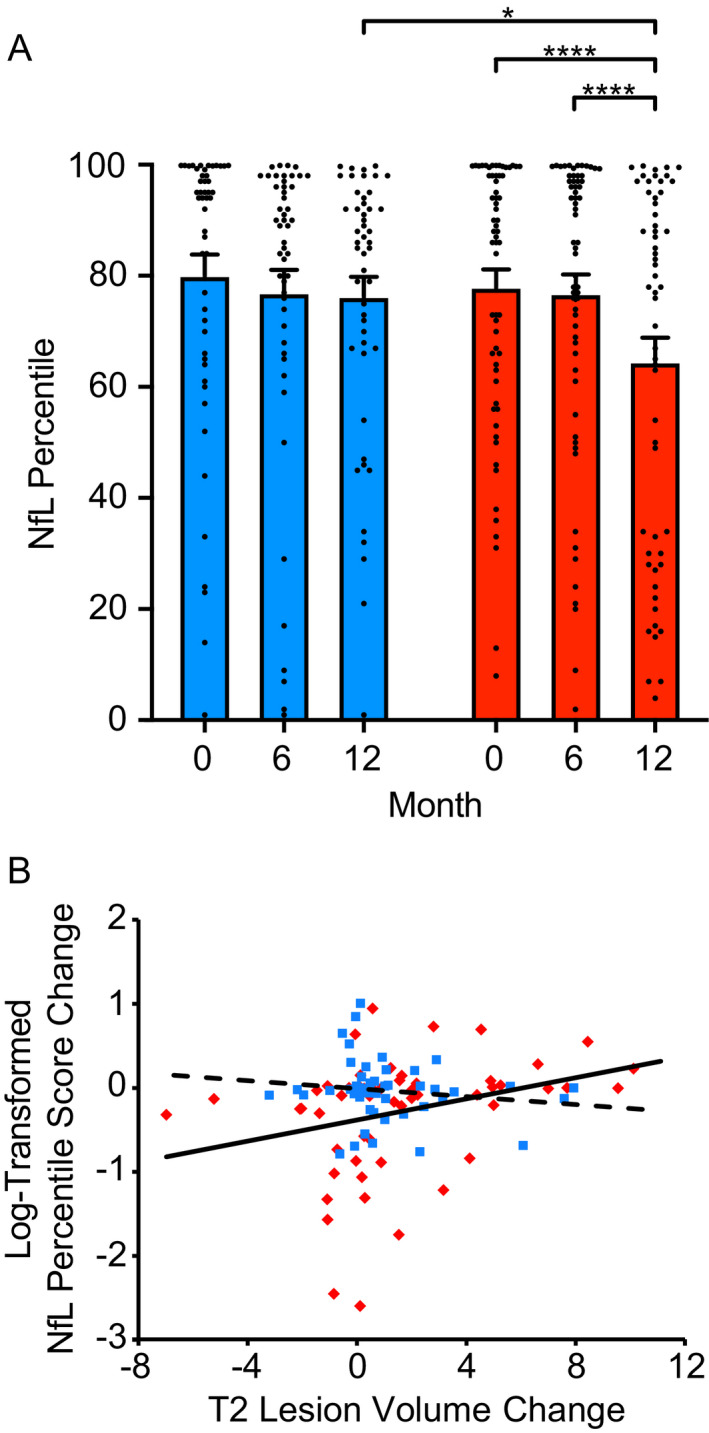
Serum NfL levels are decreased with Estriol 8 mg + GA treatment compared to Placebo + GA treatment. (A) Serum neurofilament light chain measurements (*n* = 328 samples) collected longitudinally from 111 MS patients, (62 treated with Estriol 8 mg + GA, red; 49 treated with Placebo + GA, blue) at month 0 (baseline), month 6, and month 12. The Estriol 8 mg + GA group exhibited a significant decrease in sNfL levels as compared to the Placebo + GA group. **** Indicates *p* < 0.0001, * indicates *p* < 0.05. (B) Change in log‐transformed NfL levels in the Estriol + GA group (red diamonds, solid line) was differentially associated with change in T2 lesion volume as compared to the Placebo + GA group (blue squares, hashed line). [Colour figure can be viewed at wileyonlinelibrary.com]

NfL levels were determined in serum samples from the same 111 subjects (Fig. 1A). Because NfL is a strongly age‐dependent biomarker, serum NfL concentrations are shown as age‐adjusted serum NfL expressed as a percentile.^11^, ^12^ Mixed model analyses using log‐transformed NfL percentile scores yielded a significant interaction between group and time, *F*(2,109) = 3.84, *p* = 0.02, indicating that the change in the Estriol + GA group differed significantly from the change in Placebo + GA group. There was a significant NfL decrease at 12 months for the Estriol + GA group (*t*(109) = 4.10, *p* < .0001), while the Placebo + GA group did not change significantly (*t*(109) = 0.41, *p* = 0.7) (Fig. 1A). There was no significant change in NfL from baseline to 6 months in either group. The Estriol + GA and the Placebo + GA groups differed significantly at 12 months (*t*(109) = −2.15, *p* = 0.03), but not at 6 months (*t*(109) = 0.51, *p* = 0.6).

Previously, it was shown that there was no between groups difference in T2 lesion volumes over time.^8^, ^10^ That said, mixed model analyses examining associations between changes in log‐transformed NfL percentile scores and changes in T2 lesion volume demonstrated a significant three‐way interaction between T2 lesion volume, group and time (*F*(2,109) = 3.65, *p* = 0.03), showing that the NfL change in the Estriol + GA group was differentially associated with change in T2 lesion volume as compared to the Placebo + GA group (Fig. 1B). There were no associations of change in NfL with change in EDSS, relapses, gadolinium‐enhancing lesions, or cerebral cortex volumes.

## Discussion

Estriol levels in serum of MS women were increased at both month 6 and month 12 after Estriol 8 mg treatment initiation. Reductions in sNfL levels occurred by month 12, but not by month 6. Previously, a reduction in cerebral cortical atrophy from month 0 to month 12 was observed using two complementary neuroimaging approaches: Jacobian integration and voxel‐based morphometry.^8^, ^10^ Together the decrease in sNfL and the reduction in cerebral cortex atrophy in the Estriol 8 mg + GA arm, compared to the Placebo + GA arm, is consistent with estriol‐mediated protection from neuro‐axonal injury in women with MS.^11^, ^12^, ^14^, ^15^ Since estriol binds primarily to estrogen receptor beta,^16^ this is consistent with preclinical data showing remyelination induced by estrogen receptor beta ligand treatment in MS preclinical models.^17^, ^18^, ^19^, ^20^, ^21^

The number of months of treatment required to detect a beneficial effect on MS disease biomarkers may differ for an effect of treatment on inflammation (e.g., gadolinium‐enhancing white matter lesions) versus neurodegeneration (e.g., gray matter atrophy). A previous pilot trial collected MRI data on subjects every month for 22 consecutive months to map the time course of Estriol 8 mg treatment effects on gadolinium‐enhancing lesions. Compared to the 6‐month pretreatment block, there was a reduction in enhancing lesions at 3 and 6 months after treatment.^9^ Subjects then went off estriol, and the beneficial effect on gadolinium‐enhancing lesions was partially lost over the next 3 months and fully lost by 6 months. When subjects were retreated with estriol for 4 months (in combination with a progesterone to avoid prolonged “unopposed estrogen” use), enhancing lesions again decreased. Thus, the reduction in serum NfL in the current study at month 12, but not month 6, may be less due to an anti‐inflammatory effect and more due to a neuroprotective effect. Furthermore, in the placebo‐controlled estriol treatment trial with the primary outcome measure of annualized relapse rate reduction,^8^ only 32.1% in the Estriol + GA group and 29.3% in the Placebo + GA group had enhancing lesions present on baseline MRI. Estriol treatment reduced cerebral cortex atrophy at month 12 in the subset that was gadolinium‐enhancing lesion negative at baseline (*p* = 0.043), suggesting a neuroprotective effect. Alternatively, anti‐inflammatory effects of Estriol 8 mg treatment were previously shown by cytokine changes in peripheral blood mononuclear cells^9^, ^22^, ^23^ and by the reduction in delayed‐type hypersensitivity responses.^9^ There was also a reduction in annualized confirmed relapse rate (Placebo + GA = 0.48, Estriol + GA = 0.25, *p* = 0.016)^8^ during the same 12 months that NfL levels were measured here. Beneficial effects of estriol treatment on inflammation and neurodegeneration may co‐exist and are not mutually exclusive. A limitation is the inability to distinguish between direct neuroprotective effects on neuro‐axonal injury versus indirect effects on inflammation.

In this head‐to‐head comparison, Estriol + GA treatment reduced serum NfL as early as 12 months, while Placebo + GA did not. A limitation is inability to compare serum NfL levels in estriol‐treated MS subjects with those on highly effective approved DMTs, since NfL assays here^11^, ^12^ differed slightly in methodology from that recently used to establish reference values across clinical trials.^24^ Also, heights and weights were not recorded, so the influence of body mass index (BMI) is unknown.

## Authors' Contributions

RV, PS, and AM carried out conception, design of the study, and drafting a significant portion of the manuscript or figures. JK, PS, NI, KP, and AM contributed to acquisition and analysis of data. Site investigators and co‐investigators carried out data acquisition from patients by the Estriol Trial Study Group (see Figure S1).

## Conflict of Interest

Rhonda Voskuhl is an inventor on a patent owned by the University of California, Los Angeles (UCLA) that involves estriol treatment for multiple sclerosis. Jens Kuhle, Prabha Siddarth, Noriko Itoh, Kevin Patel, and Allan MacKenzie‐Graham each have nothing to report.

## Supporting information


**Figure S1** The author contributions include acquisition of data by the Estriol Trial Study Group.
