# Supplementary material for: Decreased neurofilament light chain levels in estriol‐treated multiple sclerosis
Source: Ann Clin Transl Neurol. 2022 Jun 29;9(8):1316–20. doi: 10.1002/acn3.51622 (PMC9380170; doi:10.1002/acn3.51622)
Supplement: Supplementary file 1 — Figure S1 The author contributions include acquisition of data by the Estriol Trial Study Group. [file ACN3-9-1316-s001.docx]

**Author Contributions (Supplemental)**

Data acquisition from patients by the Estriol Trial Study Group

Jacqueline Bernard, MD, University of Chicago

John Corboy, MD, University of Colorado

Anne Cross, MD, Washington University

Suhayl Dhib-Jalbut, MD, Rutgers

Corey Ford, MD, University of New Mexico

Elliot Frohman, MD, University of Texas Southwestern

Barbara Giesser, MD, University of California, Los Angeles

Dina Jacobs, MD, University of Pennsylvania

Lloyd Kasper, MD, Dartmouth University

Sharon Lynch, MD, University of Kansas

Callene Momtazee, MD, University of California, Los Angeles

Gareth Parry, MD, University of Minnesota

Michael Racke, MD, Ohio State University

Anthony Reder, MD, University of Chicago

John Rose, MD, University of Utah

Dean Wingerchuk, MD, Mayo Clinic
